# Knowledge of Social Affiliations Biases Economic Decisions

**DOI:** 10.1371/journal.pone.0159918

**Published:** 2016-07-21

**Authors:** Joel E. Martinez, Michael L. Mack, Bernard D. Gelman, Alison R. Preston

**Affiliations:** 1 Department of Psychology, Princeton University, Princeton, NJ, United States of America; 2 Department of Psychology, The University of Texas at Austin, Austin, TX, United States of America; 3 Center for Learning and Memory, The University of Texas at Austin, Austin, TX, United States of America; 4 Department of Neuroscience, The University of Texas at Austin, Austin, TX, United States of America; Centre for Coevolution of Biology & Culture, University of Durham, UNITED KINGDOM

## Abstract

An individual’s reputation and group membership can produce automatic judgments and behaviors toward that individual. Whether an individual’s social reputation impacts interactions with affiliates has yet to be demonstrated. We tested the hypothesis that during initial encounters with others, existing knowledge of their social network guides behavior toward them. Participants learned reputations (cooperate, defect, or equal mix) for virtual players through an iterated economic game (EG). Then, participants learned one novel friend for each player. The critical question was how participants treated the friends in a single-shot EG after the friend-learning phase. Participants tended to cooperate with friends of cooperators and defect on friends of defectors, indicative of a decision making bias based on memory for social affiliations. Interestingly, participants’ explicit predictions of the friends’ future behavior showed no such bias. Moreover, the bias to defect on friends of defectors was enhanced when affiliations were learned in a social context; participants who learned to associate novel faces with player faces during reinforcement learning did not show reputation-based bias for associates of defectors during single-shot EG. These data indicate that when faced with risky social decisions, memories of social connections influence behavior implicitly.

## Introduction

Imagine buying a new car. You arrive at the dealership ready to haggle for a good deal, but discover that the salesman is a good friend of a trusted coworker. Do you act as a rational agent and drive for a hard bargain? Or does the salesman’s connection to your coworker bias your decision making, leading you to trust his first offer as the best possible deal? Recent work suggests that people’s decisions are biased by an interacting partner’s physical appearance [1,2]. Here, we focus on how social connections, specifically knowledge of a person’s friends and how they behave, bias our initial interactions with others.

Social reputation and group membership are important contextual influences on interpersonal interactions [3]. Social decisions are biased by group membership, often through complex dynamics among multiple factors [4]. For instance, automatic biases toward an individual elicited by that individual’s race can be modulated and in some cases overridden by shifts in self-categorization. Specifically, a person who identifies with a mixed-race group will show less biased interactions with other-race, in-group members compared to other-race non-members [5]. Moreover, an individual’s positive and negative reputation can push others to reward or punish that individual [6–8], even when reputation information is derived from third-party sources [9,10]. Although an individual’s reputation or group identity impacts behavior toward that individual, it remains unknown if and how these biases extend to other members of that individual’s social network. The generalization of attitude information from a target person to an associated individual is a well studied phenomenon [11–15]. Here, we extend upon existing work to examine two questions: 1) whether reputation bias extends to interpersonal decision-making and 2) whether such biases occur automatically or only when imbued with social significance. In other words, when encountering a new person, is our behavior toward the person based on our prior interactions with their friends?

We explore the possibility that associative memory is a key cognitive system that influences context-based social decision making in human interactions [16]. Hippocampal-based memory processes, well known for building detailed records of individual experiences [17,18], are implicated in encoding complex relationships among similar experiences by integrating new information with past events [19]. Such memory integration supports the dissemination of affective valence between related memories in fear conditioning [20], associative reward learning [21], and evaluative conditioning [22]. Moreover, memory systems are implicated in complex social behavior such as formation of social hierarchies [23,24], racial stereotypes [25], and the acquisition of preferences through conditioning [14,26].

These findings suggest that we access and use pre-existing knowledge when evaluating new information. In the case of interpersonal interactions, our memory for people with similar physical characteristics and social qualities (e.g., race, group membership) influences how we evaluate new people we meet [27]. For example, an individual’s moral status can affect evaluations of novel people who share facial features with that individual [28,29]. However, less is known about how pre-existing knowledge of social relationships might affect decision making. We hypothesize that during new encounters with others, people use existing knowledge of the interacting partner’s social network to guide behavior toward them. More specifically, we focus on dyadic friendships.

Dyadic affiliations are a fundamental group that serve important functional roles in daily social lives [30,31], making them ideal to study the biases that emerge from person-to-person associations. Classic research suggests that valence in friend associations should be “balanced” as a function of conscious motivational drives [32]. Here, we extend this idea by hypothesizing that knowledge about friendships can impact social interactions as result of memory integration. Previous research has shown evidence of both explicit and implicit associative transfer of attitudes [11,12,14,22]. Therefore, our knowledge about one individual’s behavior might influence how we interact with people whom we learn are their friends based on biases that arise from integrating person knowledge across social connections.

**Fig 1:**
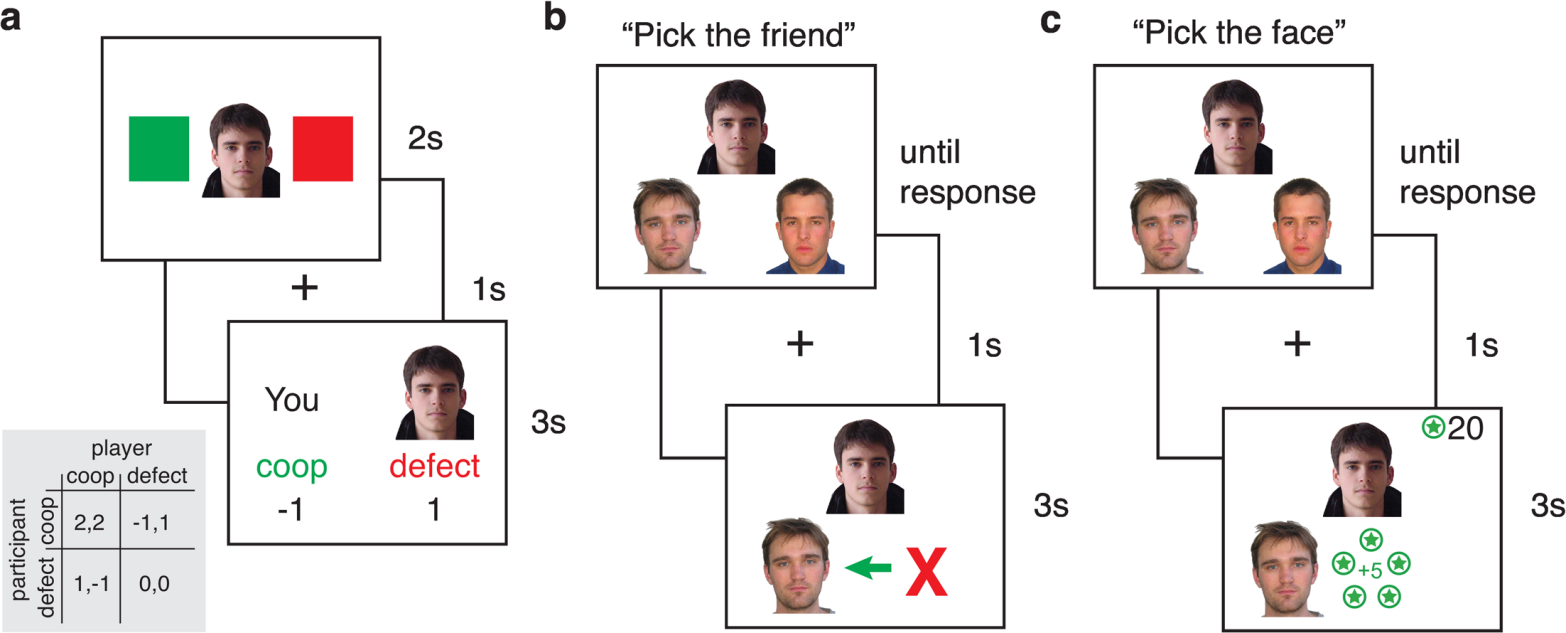
Schematic of task phases. (**a)** Economic Game task. On each trial, participants were presented with a virtual player’s image and had to choose to cooperate or defect. After a 1s fixation screen, the outcome of the trial was presented for 3s. This outcome consisted of the participant and player’s choices as well as the points earned or lost according to the payoff matrix (gray inset). (**b)** Friend association task (social framing task). Half of the participants learned associations between players form the initial EG phase and new faces in a social framing friend association task. On each trial, participants were presented with a player or novel face and instructed to choose the friend from among two faces presented below. The trial continued only after a response was made, at which point, a fixation screen was presented for 1s followed by a 3s feedback consisting of the correct friendship association. Each friendship pair was repeated six times over the course of the friend association task. (**c)** Associative face learning task (nonsocial framing task). The other half of participants learned associations between players and new faces in a nonsocial framing association task. Participants were instructed to choose the face that led to a reward without reference to a social relationship among faces. The participants’ feedback consisted of either 5 star coins for correct trials or a red X for incorrect trials. The participants’ goal was to acquire as many points as they could; accordingly, they saw a running total of their points in the upper right hand corner of the display. As in the friend association task, each face-face pair was presented six times during this phase.

To examine how knowledge about social relationship impacts decisions, we used an Economic Game (EG) that was a modified version of the classic Prisoner’s Dilemma [33]. Participants first established reputations for a set of virtual players through iterated EG trials (**Fig 1**), learning that some players typically cooperated, others defected, and some cooperated and defected equally often. Then, participants learned to associate new faces with the players through one of two tasks. In the social framing task, participants learned one novel “friend” for each of the players as well as friendships between novel people all under the guise of a different experimental goal. In the nonsocial framing task, participants learned associations between players and new faces via a reward-based reinforcement learning task that made no reference to friendships. Manipulating the framing of the associative learning tasks allowed us to test whether decision making biases based on associative knowledge occur automatically (e.g., [21,22]), as has been observed in spontaneous trait transference [34], or only when associations are learned in a social context.

The critical question is how participants treated the friends learned in the social framing task in a single-shot EG decision. If decisions about friends are influenced by the social reputation of the initial players, it is expected that participants will cooperate with friends of cooperative players and defect on the friends of defecting players. Alternatively, social reputation may not be encoded or transferred across associative memories that link player to friend. In this case, participants’ cooperative decisions with the friends would not be influenced by prior interactions with associated players. Importantly, we assessed the single-shot EG decisions for the friends learned in the social framing task relative to the single-shot EG decisions for the new faces learned in the reward-based nonsocial framing task. If memory-driven decision biases require social significance, we expected that participants would cooperate with friends of cooperators and defect with friends of defectors only when they learn the relationships as friendships in the social framing task. If general associative mechanisms underlie reputation bias, then we would expect biased decision making in both the social and nonsocial framing tasks.

## General Methods

### Participants

Sixty-four undergraduate students from the University of Texas at Austin (39 females, 20.21 years mean age) participated in the experiment that was approved by the University of Texas Institutional Review Board (#2007-09-0029). Participants provided written consent to participate in this study. This sample size was informed by an a priori power analysis from a pilot study using a similar paradigm. In this pilot study, an effect size of 0.409 (η2 = 0.143) was observed for the reputation factor in a linear mixed effects analysis of the critical transfer task described below. Power analysis of this main effect with α = 0.05 and power = 0.95 indicated a required sample size of 26. We used this estimated sample size plus our previous experience with participant exclusions in multiple day, associative learning experiments to determine our sample size. Five participants were excluded for not following the experimental instructions, four participants were excluded for failing to learn the player reputations during the iterated EG, and one participant was excluded for incomplete data. Data from the remaining participants were included in the main analyses (n = 27 for both the social and nonsocial framing task). All participants were compensated with course credit or paid $20.

### Stimuli

The stimuli were colored images of 96 Caucasian, college-aged faces (half female and half male) gathered from the Computer Vision Laboratory, University of Ljubljana, Slovenia [35,36]. Faces were previously normed for neutral facial expressions. Stimuli had dimensions of 500 x 400 pixels and subtended approximately 12° x 10° visual angle.

### Procedure

The experiment was implemented using Matlab with Psychtoolbox and run on Apple iMac computers. The experiment consisted of two experimental sessions scheduled approximately 24 hours apart.

### Day 1: Iterated Economic Game

To establish social reputations, participants played an iterated EG game with a set of 24 virtual “players”. Participants were instructed that 1) they would play a game in which they would decide to cooperate with or defect on other players to earn points, 2) point outcomes were contingent on the other players’ choices and followed the rules of a payoff matrix (**Fig 1A** inset), and 3) their goal was to maximize their own point total, regardless of how many points the other players received. Participants were also told that the other players’ choices were based on behavioral profiles of choice probabilities from past participants who had completed the experiment. Participants were further instructed that their compensation would not be linked to performance. The payoff matrix was modified from a standard Prisoner’s Dilemma matrix to encourage reciprocity in behavior. Our goal was to maximize participants’ ability to learn the social reputation of players. Accordingly, we set a payoff matrix for which the optimal strategy was to cooperate with cooperative players and defect against defectors.

On each trial (**Fig 1A**), participants were presented with an image of a player for 2s. During this time, participants chose to cooperate or defect by pressing the ‘1’ or ‘2’ key. After a 1s fixation delay, feedback of the EG outcome along with the player’s image was presented for 3s. Feedback consisted of the participant’s response, the player’s response, and the points earned or lost by both the participant and player.

Participants played against each of the 24 virtual players in mini-blocks consisting of six consecutive EG trials with the same player. The virtual players were split into three reputation conditions: cooperators, defectors, and 50/50. During a mini-block, cooperators randomly cooperated during 5 trials (83.3%), defectors randomly cooperated in 1 trial (16.7%), and 50/50 players randomly cooperated in 3 trials (50%).

Across the experiment, participants experienced 2 mini-blocks with each of the 24 virtual players, for a total of 48 mini-blocks and 288 trials. The 48 mini-blocks were split into 4 larger blocks consisting of 12 mini-blocks with a brief break between blocks. Face stimuli were randomly assigned to different reputation conditions across participants. The order of the mini-blocks was also randomized across participants.

### Day 1: Associative learning of face pairs

Participants learned to associate one new face with each player from the initial iterated EG phase in one of two tasks. In the *social framing task*, the face associations were framed in terms of their social significance. In contrast for the *nonsocial framing task*, the face associations were framed as a reward contingency. Comparison of the findings from these two learning conditions therefore allowed us to examine whether reputation bias results from a general associative mechanism [21,34], or is a uniquely social phenomenon. Half of the participants performed the social framing task and the other half performed the nonsocial framing task. The specific similarities and differences between these tasks are detailed below.

In both tasks (**Fig 1B and 1C**), participants learned associations between the 24 virtual players encountered during the iterated EG and 24 novel faces, as well as associations between 48 novel faces not previously seen (no reputation condition) through trial and error learning. Each player and no reputation face was paired with a single novel face. Face pairs were selected to balance gender combinations within all conditions. Participants were instructed that this task was separate from the initial iterated EG task under the guise of a different experimental goal.

The social and nonsocial framing tasks both consisted of a two-alternative forced choice task with feedback. On each trial, participants were shown a cue face at the top center of the screen (either a player from the iterated EG phase or a new, no reputation face) and two choice faces on the bottom left and right (both novel). The critical difference in the tasks was their framing. In the social framing task, participants were instructed to “Pick the friend” with the goal of maximizing accuracy for friendship associations. In contrast, in the nonsocial framing task, no mention was made of social relationships between the face pairings. Instead, participants were instructed to “Pick the face” that led to a reward of 5 star coins, with the overall goal of maximizing points accrued across this associative training phase. Accordingly, in the nonsocial framing task, participants saw a running total of earned star points at the top right corner of the screen. In both tasks, participants selected which of the two bottom faces was associated with the top face by pressing the “1” or “2” key for the left or right face, respectively. Distractor faces remained consistent across repetitions of a given association. Participants had unlimited time to make a response, after which a 1s fixation screen was presented and followed by a 3s feedback screen. During the feedback phase, the cue face was displayed along with the correct face. Participants also received feedback regarding whether or not their response was correct. Participants completed 6 blocks, with each block consisting of one trial for each of the 48 friend pairs. Trials were presented in randomized order within each block.

### Day 2: Critical reputation transfer task

Approximately 24 hours after the associative learning task, participants returned to complete the critical testing phase. Participants first played a randomized one-shot EG with the 48 face associates they encountered in the friend or face learning phase on Day 1. The 48 associates included faces paired with players from the iterated EG phase on Day 1 as well as faces paired with new people (no reputation condition). No reference was made to the prior day’s events either in the instructions or by the experimenter. Participants were told their goal was to maximize the number of points received. The one-shot EG task followed the same procedure as the iterated EG with the following exceptions: 1) participants had unlimited time to make a response, 2) no feedback was given, and 3) each of the 48 associates were presented only once. The trial order for this phase was randomized across participants. After making their own response on each trial, participants were also asked to make a prediction about what move they thought the player selected on that trial. This prediction measure was highly consistent with participants’ one shot EG decision in both the social (*rs*(25) > 0.30, *ps* < 0.12) and non-social framing conditions (*rs*(25) > 0.60, *ps* < 0.0001) for each of the four reputation types. In other words, if a participant chose to cooperate on a trial, they were likely to indicate that they thought the other individual chose to cooperate as well. Because of the high correlation between these indices, we focus our reporting solely on participant choices from the one shot EG phase.

### Day 2: Memory test for the face associations

After the critical transfer task, participants completed a surprise memory test for the 48 pairs learned on Day 1. The task instructions were identical to the task-specific memory test on Day 1 (**Fig 1B and 1C**), with the exception that no feedback was given.

### Day 2: Explicit predictions regarding future behavior

Next, we administered a cooperative prediction measure for the associates of the players and the players in the cooperator, defector, 50/50, and no reputation conditions. First, participants were asked the following question for each associate, “If you met this person 100 times in the future, how many of those 100 times would they cooperate?” The associated face was displayed in the center of the screen below the question text. Responses were self-paced and made by typing a number ranging from 0 to 100. A 1s ITI separated each trial. Second, participants were asked the same prediction question about each of the players. Following each prediction, participants also categorized each player into a reputation type based on the player’s behavior from the iterated EG. Participants were given definitions of the reputation type categories: a cooperator was someone who cooperated most of the time in the iterated EG, a defector was someone who defected most of the time, and 50/50 was someone who didn’t favor one response over the other. Category responses were made by pressing the “1” key to indicate a cooperator, “2” key to indicate a defector, and the “3” key to indicate a 50/50 response. All of the judgments were self-paced and a 1s ITI separated the questions.

## Analysis

Behavior was analyzed with a combination of linear and logistic mixed effects models and planned comparisons. We first assessed acquisition of player reputations during the iterated EG. We constructed three models for this analysis: two models assessing performance in the social and nonsocial framing tasks and an additional model examining the interaction between task framing and the variables of interest. The main factors in these models were reputation condition and mini-block, with participants as a random factor. We further included random slopes for reputation condition, mini-block, and their interaction in each of the models.

The second analysis focused on performance in the explicit categorization task. Again, we constructed three models: one each for the two different framing tasks and an additional model assessing the interaction of framing and the variables of interest. The main factors in this model were reputation condition and category response; random factors included participants and the slopes for category response and reputation condition.

We also created models assessing performance in the association learning, memory tasks, and the one-shot EG. For each of these models, reputation condition served as the main predictor, with participants as a random factor. As with the other analyses, three models were constructed for each behavioral metric that characterized performance in the two framing conditions, with a third model assessing the interaction between the framing conditions. We also performed logistic regression to quantify performance in the one-shot EG, using the binary trial data with reputation condition as a varying slope.

Reported results are based on type III summed square errors of the factors with Satterthwaite approximations of degrees of freedom. Cohen’s *f*^*2*^ effect sizes are reported for regression model results. Odds ratios (OR) are reported for the logistic regression. Planned comparisons were conducted using the “lsmeans” R package [37].

## Results

### Establishing Player Reputations

**Fig 2:**
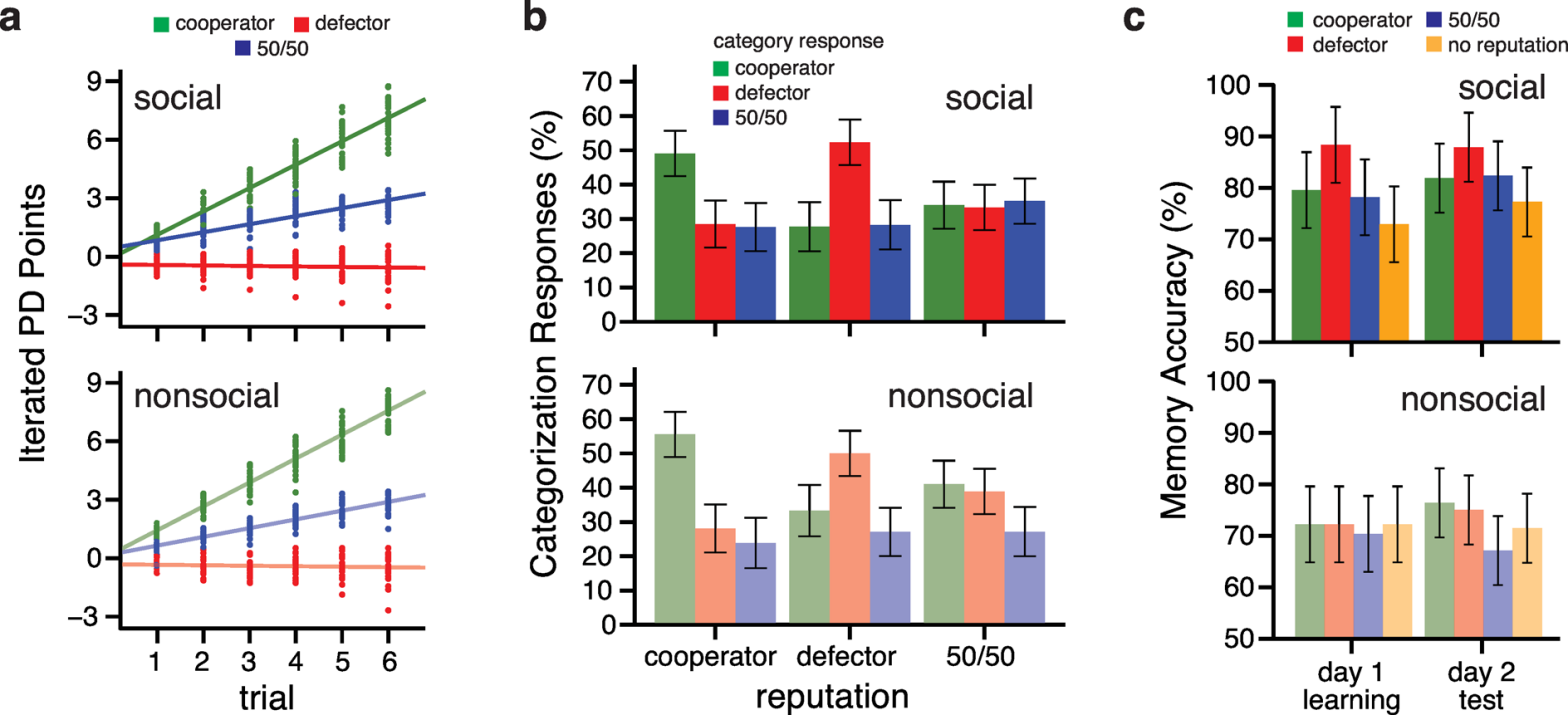
Establishing reputations and memory of associations for the social (saturated) and nonsocial framing groups (lighter). (**a)** Average points won during the six trials within a mini-block during the iterated EG task separated by player type (cooperator–green, defector–red, and 50/50 –blue). Points represent individual subject point totals and solid lines depict the player type regression lines from the linear mixed effects model. (**b)** Average percentage of category responses during the categorization task on day 2 separated by actual player type and further by categorized player type. Error bars represent 95% confidence intervals of the mean. **c)** Average associative memory performance at the end of day 1 learning (left bars) and day 2 memory test (right bars) separated by reputation condition type (cooperator–green, defector–red, 50/50 –blue, no reputation–yellow). Error bars represent 95% confidence intervals of the mean.

Participants first played iterated EG with each player to establish the players’ reputations for cooperating or defecting. We quantified participants’ behavior in this phase by comparing the average total points earned across a mini-block (see **Fig 2A**). We calculated average points earned for the three player reputation types during each of the six consecutive trials of a mini-block. For the social framing group, we observed a significant reputation by block interaction (*F*_*2*,*27*_ = 464.69, *p* < 0.0001, *f*^*2*^ = 0.204). Participants consistently earned more points when playing against cooperators (slope = 1.200, 95% CI = [1.142, 1.258]) than 50/50 players (slope = 0.413, 95% CI = [0.377, 0.448]) or defectors (slope = -0.026, 95% CI = [-0.074, 0.023]). We observed similar behavior with the nonsocial framing group, with a significant interaction of reputation by block (*F*_*2*,*27*_ = 539.5, *p* < 0.0001, *f*^*2*^ = 0.403); the most points were gained on cooperator trials (slope = 1.228, 95% CI = [1.169, 1.286]), followed by 50/50 player (slope = 0.449, 95% CI = [0.413, 0.483]) and defector trials (slope = -0.025, 95% CI = [-0.073, 0.023]). There was no interaction between block, reputation, and framing (*F*_*2*,*54*_ = .300, *p* = .719, *f*^*2*^ = .0002); thus, performance during the iterated EG did not differ across the framing tasks. These point outcomes reflect optimal strategies against each reputation type based on the payoff matrix and confirmed that participants learned the players’ reputation without explicit instruction to do so. In other words, participants cooperated with cooperators, defected on defectors, and dynamically shifted their performance with 50/50 players.

This knowledge of player reputation persisted to the second day of the experiment, as indicated by participants’ categorization of players at the end of the experiment (**Fig 2B**). For both framing groups, we observed a significant interaction of reputation condition and category response (social: *F*_*4*,*176*_ = 16.84, *p* < 0.0001, *f*^*2*^ = 0.382; nonsocial: *F*_*4*,*168*_ = 14.18, *p* < 0.0001, *f*^*2*^ = 0.357), reflecting the tendency for participants to correctly categorize cooperators and defectors. There was no interaction between categorization, reputation, and framing (*F*_*4*,*344*_ = 0.857, *p* = 0.490, *f*^*2*^ = 0.011), indicating categorization performance was similar across the two framing tasks.

### Learning Face Associations

After completing the iterated EG, participants learned to associate a new face with each player through trial-and-error learning as well as novel faces pairs with no reputation. The context of this learning differed across framing groups, such that the social framing group learned one “friend” for each player and the nonsocial framing group learned the face associations through rewards. To assess associative learning, we evaluated memory performance for face associations on the last learning repetition (**Fig 2C**). We found a significant reputation by framing context interaction in memory performance (*F*_*3*,*162*_ = 3.636, *p* = 0.0142, *f*^*2*^ = 0.067). For the social framing group, memory performance differed across reputation condition (*F*_*3*,*81*_ = 9.080, *p* < 0.0001, *f*^*2*^ = 0.337) such that associations with defector players were better remembered than the other associations (*t*s(168) > 2.604, *p*s < 0.0101). In contrast, we observed no differences in memory performance across reputation for the nonsocial framing group (*F*_*3*,*81*_ = 0.133, *p* = 0.940, *f*^*2*^ = 0.005). Moreover, comparisons across the framing groups for the separate reputation conditions showed that participants in the social framing group remembered defector associations better than participants in the nonsocial framing group (*t(110)* = 3.08, *p* = 0.0026), with no group difference in memory for the other reputation conditions (*t*s < 1.495, *p*s > 0.138).

The memory advantage for the defector friend associations in the social framing group remained on the second day (**Fig 2C**). In particular, the social framing group showed a memory advantage over the nonsocial framing group for defector (*t(132)* = 2.70, *p* = 0.0078) and 50/50 player (*t(132)* = 3.19, *p* = 0.0018) associations, but no difference for cooperator and no reputation associations (*t*s(132) < 1.22, *p*s > 0.22). These findings suggest that although both framing groups had similar baseline associative memory, as measured in the no reputation condition, social framing improved memory for associations involving players that demonstrated negative social behavior.

### Social relationships implicitly bias decision making

**Fig 3:**
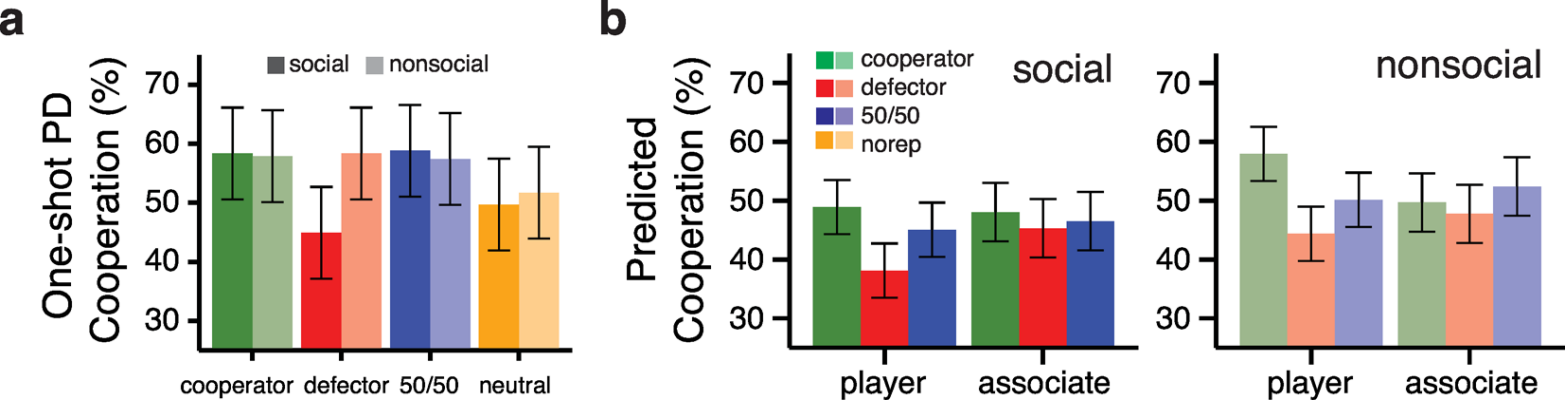
Cooperation behavior in one-shot EG and explicit predictions of cooperative behavior. (**a)** Average percentage of cooperative plays with associated faces during one-shot EG separated by the associated player reputation type for the social (saturated colors) and nonsocial (lighter colors) framing groups. (**b)** Average predicted cooperation in future EG games for players and associated faces separated by framing group. Error bars represent 95% confidence intervals of the mean.

On the second day, participants played a one-shot EG with the faces learned in the associative memory phase from the first day. Behavior in the social framing group, who learned the associated faces as friends of the players, was influenced by the reputation of the player associated with each friend (**Fig 3A**; main effect of friend reputation: *F*_*3*,*81*_ = 5.456, *p* = 0.0018, *f*^*2*^ = 0.203). Participants in this group were less likely to cooperate with friends of defectors than friends of cooperators (OR = 0.57, 95% CI = [0.37, 0.86], *t(168)* = 3.18, *p* = 0.0018) and friends of 50/50 players (OR = 0.55, 95% CI = [0.36, 0.84], *t(168)* = 3.29, *p* = 0.0012). They were also less likely to cooperate with friends in no reputation condition than friends of cooperators (OR = 0.69, 95% CI = [0.50, 0.97], *t(168)* = 2.05, *p* = .042) and friends of 50/50 players (OR = 0.67, 95% CI = [0.48, 0.94], *t(168)* = 2.16, *p* = .033), but not friends of defectors (OR = 1.22, 95% CI = [0.88, 1.70], *t(168)* = 1.13, *p* = .259).

However, behavior in the nonsocial framing group differed from participants in the social framing context (*F*_*3*,*162*_ = 2.72, *p* = 0.046, *f*^*2*^ = 0.051). The nonsocial framing group, who learned face associations through reward-based feedback, showed no evidence for reputation-based bias in one-shot EG behavior (*F*_*3*,*81*_ = 1.11, *p* = 0.350, *f*^*2*^ = 0.041). Importantly, the lack of reputation bias in the nonsocial framing group did not result from differences in learning face associations, as there was no evidence for reputation bias even when the analyses were restricted to remembered face associations (*F*_*3*,*78*_ = 0.56, *p* = 0.641, *f*^*2*^ = 0.001). Comparisons between the framing groups showed that reputation bias in social framing group was specific to defector associations (OR = 1.84, 95% CI = [1.10, 3.09], *t(145)* = 2.41, *p* = 0.017); there were no group differences in one shot EG behavior for faces associated with cooperators, 50/50 players, or no reputation faces (*t*s(145) < 0.36, *p*s > 0.72).

Interestingly, social framing participants did not demonstrate an explicit bias to treat friends according to the reputations of associated players. Whereas predictions about players in future games differed according to reputation (Fig 3B; *F*_*2*,*54*_ = 8.659, *p* = 0.0006, *f*^*2*^ = 0.322), predictions about friends showed no bias (*F*_*2*,*54*_ = 1.199, *p* = 0.309, *f*^*2*^ = 0.045). Participants in the nonsocial framing group made predictions about players’ (*F*_*2*,*54*_ = 16.663, *p* < 0.0001, *f*^*2*^ = 0.621) and associates’ (*F*_*2*,*54*_ = 4.942, *p* = .011, *f*^*2*^ = 0.183) future behavior based on the players’ reputation. The effect of reputation on associate predictions was driven by a tendency to predict faces associated with 50/50 players as being more cooperative, with no differences in predictions between associates of cooperators and defectors (*z* = 1.645, *p* = 0.103). Moreover, predictions for players *(F*_*2*,*108*_ = .657, *p* = 0.520, *f*^*2*^ = 0.012) and associated faces (*F*_*2*,*108*_ = 1.905, *p* = 0.154, *f*^*2*^ = 0.035) did not differ between the two framing groups. These findings suggest that participants’ explicit judgments about the future behavior of individuals associated with players were not influenced by player reputation.

## Discussion

Our findings provide empirical evidence that individuals’ memory of social relationships influences how they interact with new people. We targeted the influence of social relationships on decision making with an economic game, finding that participants behaved with a marked bias for the reputation of a person’s affiliates when relationships were framed in terms of their social significance. Knowledge about social affiliations led participants to defect with friends of defectors more so than when associations between people were learned in a non-social context. The reputation-based bias in the social framing group did not track participants’ explicit judgments of how the friend would play in the future, suggesting that the bias did not arise from an explicit strategy. Moreover, there was no evidence of reputation-based bias when associations between people were learned according to nonsocial reward contingencies. These findings indicate the social specificity of the reputation bias across associated individuals, differentiating it from general associative phenomena such as spontaneous trait transference [34].

Previous work has proposed that heuristics unconsciously guide inferences in social situations [38]. The automatic processing of social cues may lead to both favorable (e.g., charity [39]) and harmful (e.g., prejudice, [29]; stereotypes, [5])[5,29] interpersonal outcomes. We extend this work by characterizing the development of heuristics originating from dyadic associations. Heuristics often arise from first party information, such as a direct observation or past experience. For instance, people automatically use facial similarity to known individuals as a predictive cue in encounters with novel people [40]. Our findings build upon this work and indicate that past experience with an individual’s close affiliates is also influential. The reputations of an individual’s friends do not necessarily reflect characteristics of the individual. However, their relevance as informative contextual cues may be elevated in the absence of direct behavioral information. The present findings support the idea that the unconscious is an adaptive behavioral guidance system, sensitive to social context [41] when uncertainty is high due to a lack of information [42].

Consistent with past research on social exchange, participants learned and remembered the players’ reputations after repeated interactions and without explicit instruction [43,44]. In the social framing task, this memory advantage was stronger for players with clear positive or negative reputations, in line with the idea that people instinctively form robust evaluations through reputational [44] or emotional [45] categorization of others. Thus, remembering behavioral patterns is beneficial [46], and automatically encoding social behavior is an important source for heuristic development.

Prior work has identified various inferential biases in decision making. For instance, value can be transferred between associated items, unconsciously influencing preferences for items with no directly learned value [21]. Similarly, social attitudes toward a person can transfer implicitly to associated individuals in the absence of explicit transfer [11,12,38,47,48]. We extend these findings to show that social value (here cooperative reputation) implicitly influences interactions with social affiliates of previously encountered individuals by biasing decisions but not predictions. Our findings thus indicate that decision making during interpersonal interactions is dissociable from social judgments in some cases.

One possible interpretation of our findings is that reputation is transferred across affiliates through a memory integration mechanism [19] that links new information to preexisting knowledge. This mechanism predicts that new experiences (i.e., friend A is paired with player B) triggers retrieval of related knowledge (i.e., player B typically cooperates). By reactivating prior memories during new learning, relational memory networks are formed that span similar experiences and may lead to the transfer of social reputation from one individual to another (i.e., friend A also cooperates). Consistent with this proposed memory integration mechanism, we found that reputation biases in economic decisions were often mirrored by biases in associative memory. Notably, participants had better memory of player-friend associations for defectors over other reputations when learned with a social framing, consistent with the notion that behaviors with negative outcomes have a mnemonic priority [49,50]. Thus, the condition for which we observed the largest effect of reputation on economic decisions—defectors in the social framing task—was notably the condition for which memory was enhanced in the social framing relative to the nonsocial framing group, suggesting a link between memory behavior and bias in social decision making.

Alternatively, it is possible that reputation information itself is not bound to social affiliates; rather, participants may have been concerned for their own reputation when interacting with friends, focusing on how their behavior on a given trial might influence future payoffs [51]. For instance, someone who has cooperated with you in the past may continue to cooperate in the future if they learn you have treated their friend well. In other words, interactions with new individuals in the present task may have served as a proxy interaction with the known player. This account of the findings is not mutually exclusive with the reputation transfer interpretation discussed above, and it is likely that both mechanisms influence behavior in the present task. Importantly, even the proxy interaction account of our data relies on associative memory mechanisms that allow interactions with related individuals to mutually influence one another.

Memory integration could also underlie other social phenomena, such as prejudice and stereotyping, in which physical features lead to automatic reactivation of mental models about the individual’s identified group [15]. A recent account even showed that racial biases could be unlearned through memory reactivation during sleep [52]. Evidence suggests these relational memory processes are mediated by hippocampal-based memory networks [53] that have also been implicated in the transfer of affective valence in fear conditioning [20], associative reward learning [21], and in the formation of social hierarchies [23]. An intriguing avenue for future research is characterizing the role of hippocampal-dependent memory systems in the flexible encoding of social information.

Further evidence for the importance of memory in reputation transfer comes from the critical role context can play in interpersonal interactions. For example, one can flexibly evaluate an individual by their race, age, or gender depending on the environment in which they are encountered [54]. In the current study, the social and nonsocial learning context differed in the amount of reputation bias observed; excluding the social framing when participants learned the face associations eliminated the reputation bias. This finding suggests that the reputations of the players were not accessed when the associative learning task shifted attention away from the social significance of the relationships among faces. Thus, new individuals were linked to reputation information about the associated players. In contrast, learning the face associations in a friendship context encouraged participants to treat the stimuli as social entities, thereby promoting links between existing knowledge about the reputation of the player and their friends. This finding suggests that social information needs to be contextually relevant to influence the associative learning process, and that social and reward-based associative learning do have different consequences for behavior [16].

It is worth noting two additional effects in our results. In contrast to their superior associative memory performance for defector friendships, the social framing participants were equally likely to correctly categorize defector and cooperator players according to their learned reputation (*z* = 0.732, *p* = 0.464; **Fig 2B**). Thus, although these participants remembered the players’ reputations regardless of whether that reputation was positive or negative, the friendships of defectors were more quickly learned. Prior work has shown similar selective benefits for remembering individuals with negative reputations. For example, source memory for cheaters (i.e., the context of the cheating) is enhanced relative to source memory for trustworthy individuals, whereas old/new recognition memory is equivalent for both reputations [55]. Such selective mnemonic benefit is thought to arise from emotional reactions to cheating during encoding [56]. A similar mechanism may be at play in our task; the negative emotional reaction to the defectors may linger during friend learning and strengthen the associative memory formed for the friendship. The lack of a reputation effect on associative memory with a nonsocial framing further supports the notion that social framing guides the way in which memories are constructed, leading to subsequent behavioral biases.

Second, in the critical transfer task for the social framing group, participants treated friends in the no reputation condition more like friends of defectors than friends of cooperators or 50/50 players. Without reputation information to guide behavior, participants defaulted to cautiously defecting more. Accessible social information can affect trust decisions in economic games: positive descriptors increase cooperation, while negative descriptors increase rejections [42]. However, people are generally risk averse when making decisions involving potential gains and losses [57]. The payoff matrix in our task led to equivalent point outcomes for cooperating and defecting, but only cooperation ran the risk of point loss. It may be that without social information in the no reputation condition, participants in our task were loss averse.

More generally, risk aversion possibly manifests as a mistrust of “strangers” in the context of social-economic decisions due to lack of social information. Conversely, it is not always adaptive to attend solely to negative information [44]. In our study, participants tended to cooperate more with friends of 50/50 players that were categorized incorrectly as cooperators (63.3%) than those that were categorized as defectors (52.8%). This finding suggests that enough positive social experience with players who have mixed reputations might decrease risk aversion. Another possibility is that the payoff matrix incentivized a cooperation strategy overall, leading participants to cooperate more often with 50/50 players. Notably, even in the face of such a cooperation incentive, participants in the social framing condition choose to defect against friends of defectors, indicating the strength of reputation bias in this condition.

In conclusion, the present findings suggest that when faced with risky decisions, memories of social connections automatically influence behavior. Returning to the car dealership, the salesman’s connection to your coworker may implicitly bias you to drive off the lot with a new car for a bad price. In the task we explored, this bias was adaptive, leading to higher potential rewards than acting otherwise. However, the transfer of reputation across social networks may not always lead to positive outcomes; such a mechanism may underlie negative implicit social behaviors including prejudice and stereotyping [15].
